# Limited polymorphism of the functional MHC class II B gene in the black‐spotted frog (*Pelophylax nigromaculatus*) identified by locus‐specific genotyping

**DOI:** 10.1002/ece3.3408

**Published:** 2017-10-20

**Authors:** Hong‐Yi Liu, Fei Xue, Jie Gong, Qiu‐Hong Wan, Sheng‐Guo Fang

**Affiliations:** ^1^ The Key Laboratory of Conservation Biology for Endangered Wildlife of the Ministry of Education, and State Conservation Centre for Gene Resources of Endangered Wildlife College of Life Sciences Zhejiang University Hangzhou China; ^2^ Co‐Innovation Center for Sustainable Forestry in Southern China College of Biology and the Environment Nanjing Forestry University Nanjing China

**Keywords:** black‐spotted frog, major histocompatibility complex, population genetic structure, purifying selection

## Abstract

Amphibians can be more vulnerable to environmental changes and diseases than other species because of their complex life cycle and physiological requirements. Therefore, understanding the adaptation of amphibians to environmental changes is crucial for their conservation. Major histocompatibility complex (MHC) presents an excellent tool for the investigation of adaptive variations and the assessment of adaptive potential because it can be under strong diversifying selection. Here, we isolated the MHC class II B (MHCIIB) gene from cDNA sequences of the black‐spotted frog (*Pelophylax nigromaculatus*), a widespread amphibian species in China, and designed locus‐specific primers to characterize adaptive variability of this amphibian. Ten alleles were identified from 67 individual frogs of three populations and no more than two alleles were present in each individual animal. Furthermore, none of the sequences had indels or/and stop codons, which is in good agreement with locus‐specific amplification of a functional gene. However, we found low polymorphism at both nucleotide and amino acid levels, even in the antigen‐binding region. Purifying selection acting at this locus was supported by the findings that the *d*_N_/*d*_S_ ratio across all alleles was below 1 and that negatively selected sites were detected by different methods. Allele frequency distributions were significantly different among geographic populations, indicating that physiographic factors may have strong effect on the genetic structure of the black‐spotted frog. This study revealed limited polymorphism of three adjacent black‐spotted frog populations at the functional MHCIIB locus, which may be attributed to region‐specific differences. The locus‐specific genotyping technique developed in this study would provide a foundation for future studies on adaptive divergence among different frog populations.

## 1 INTRODUCTION

As the transition from aquatic to terrestrial habitats caused dramatic evolutionary changes in the animal kingdom, amphibians represent an interesting model to investigate selective genetic mechanisms underlying the water‐to‐land adaptation. Thus, studies of amphibious vertebrates would elucidate the functional demands of two very different habitats and further our understanding of the initial evolutionary challenges associated with moving to dry land. Most amphibians lay eggs in water and their larvae go through a water‐inhabiting period. Because of the complex life cycle, amphibians are much more susceptible to environmental changes (Kueneman et al., 2014; Murray & Hose, 2005), which accounts for sharper decline in the amphibian population compared to other animal groups, arousing a deep concern of conservation biologists (Bauer, Paton, & Swallow, 2010; Brühl, Schmidt, Pieper, & Alscher, 2013). In order to develop strategies for the prevention of further decline in amphibian populations, it is important to understand adaptive variations and environmental adaptability of this animal class (Lillie et al., 2015; Rogell, Thörngren, Laurila, & Höglund, 2010; Zeisset & Beebee, 2010).

Major histocompatibility complex (MHC) encoded by a family of genes present in vertebrates has become an excellent tool for assessing genetic variations caused by environmental challenges (Sommer, 2005). The conspicuous variability is thought to be maintained by diversifying selection, when the main selective pressure is exerted by host interaction with different pathogens (Consuegra et al., 2013; Hauswaldt, Stuckas, Pfautsch, & Tiedemann, 2007; Shu, Hong, Yang, & Wu, 2013; Yu, Zheng, Zhang, Shen, & Dong, 2014). Each MHC molecule can present a limited set of peptides to T cells, triggering a corresponding immune response, given that the bound peptides are nonself. An individual heterozygous for MHC loci or carrying new alleles would express larger or new repertoire of MHC molecules, and thus, would have an advantage of better protection against pathogen invasion (Penn, Damjanovich, & Potts, 2002; Takahata & Nei, 1990). Therefore, MHC genetic variability is closely related to fitness and adaptability of vertebrates (Sommer, 2005).

Many studies showed that the recent emergence of several infectious diseases caused by bacteria, fungi, and parasites played a significant role in the decline of the amphibian population (Kelehear, Brown, & Shine, 2011; Koprivnikar & Redfern, 2012; Ramsey, Reinert, Harper, Woodhams, & Rollins‐Smith, 2010; Yu et al., 2014). There is increasing evidence of the association between MHC class II genetic polymorphism and the efficiency of immune protection against new diseases (Ren et al., 2011; Savage & Zamudio, 2011; Yu et al., 2014). Therefore, the research on MHC class II genes would contribute to understanding acquired immunity in amphibians and may help in developing strategies to prevent the decrease in their numbers worldwide (Shu et al., 2013; Zeisset & Beebee, 2010). MHC class II molecules are membrane‐bound heterodimeric glycoproteins consisting of the α and β chains encoded by the MHC class II A (MHCIIA) and B (MHCIIB) genes, respectively. Among them, MHCIIB is shown to exhibit higher polymorphism in many animals (Hauswaldt et al., 2007; Ren et al., 2011). Recently, MHCIIB genes have been characterized in several amphibian species (Bataille et al., 2015; Hauswaldt et al., 2007; Lillie et al., 2015; Savage & Zamudio, 2011; Shu et al., 2013; Yu et al., 2014; Zeisset & Beebee, 2010). However, locus‐specific genotyping was only implemented in a few amphibian species, while most members of the highly diverse amphibian class were not investigated, accounting for gaps in the knowledge about MHC polymorphism in these animals (Savage & Zamudio, 2011; Savage & Zamudio 2016).

**Figure 1:**
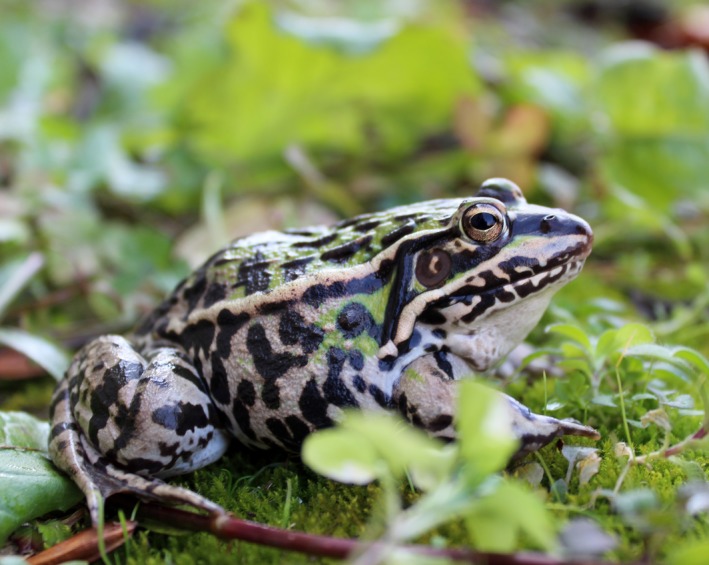
An adult black‐spotted frog (*Pelophylax nigromaculatus*). The photograph was taken by Hong‐Yi Liu

The black‐spotted frog (*Pelophylax nigromaculatus*; Figure 1) belonging to the *Pelophylax* genus inhabits both Oriental and Palaearctic realms and is widely spread across East Asia (Du, Yan, & Zhou, 2012). Although these frogs are still found in Northeast, North, East, Central, and Southwest China, their numbers are decreasing, which epitomizes the population status of many amphibians in the current ecological conditions (Du et al., 2012; Gong, Sun, Xue, Fang, & Wan, 2013). Accordingly, the black‐spotted frog can be an ideal representative species for investigating adaptation of amphibians, especially of those belonging to the *Pelophylax* genus. As most amphibians, the black‐spotted frog does not grow in captivity, and thus, may faithfully reflect the responses to the changes in the natural habitats. Considering that the information on the molecular structure of MHC in the *Pelophylax* genus is scarce, this *P*. *nigromaculatus* presents a valuable model for developing locus‐specific genotyping technique to evaluate the adaptive genetic variability of natural frog populations.

In this study, we characterized the functional MHCIIB locus in the black‐spotted frog using our locus‐specific genotyping strategy. First, DNA walking was employed to amplify complete exon 2 and partial intron 1 and 2 sequences of this locus. Then, primers for PCR‐single‐strand conformation polymorphism (SSCP) analysis were designed to examine MHCIIB variants in three black‐spotted frog populations separated by the Yangtze River. Finally, genetic structure, natural selection, and phylogenetic relationships were evaluated. To the best of our knowledge, this study is the first to identify the MHCIIB gene in the black‐spotted frog. The obtained data on frog MHC variability and the described locus‐specific genotyping strategy would provide the foundation for studying adaptive divergence among different frog populations in the future.

## 2 MATERIALS AND METHODS

### 2.1 Animals

To characterize the MHCIIB gene in the black‐spotted frog, we analyzed 67 samples acquired from three frog populations. Frogs from two populations inhabiting the south bank of the Yantze River were collected to the south of Yibin (YBS, *n *=* *23) and Luzhou (LZS, *n *=* *31), and the other frogs were collected to the north of Yibin (YBN, *n *=* *13) located at the opposite bank of the Yantze River. A noninvasive sampling method was adopted. Frog toes were sampled and kept in 95% ethyl alcohol. Frogs were released after sampling with the exception of a dying one which was preserved in liquid nitrogen and used for RNA isolation. Total RNA was extracted from the frog liver using TRIzol reagent (Invitrogen) following the manufacturer′s instructions, and first‐strand complementary DNA (cDNA) was synthesized using the PrimeScript 1st Strand cDNA Synthesis Kit (TaKaRa). Genomic DNA (gDNA) was extracted from frog toes using the phenol–chloroform protocol. Nucleic acid samples were stored at −20°C for further studies.

### 2.2 Isolation of exon 2 and introns 1 and 2 of the MHCIIB gene

Nucleic acid samples from the dying frog were used to identify exon 2 and introns 1 and 2 of the MHCIIB gene. Partial exon 2 sequence was obtained from the cDNA template of the liver using primers MHC‐AN‐3F (5′‐GGGTCAGTGTTATTACCGGAACGG‐3′) and MHC‐XEN‐M1‐R (5′‐TCCCACATCRCTGTCRAAGT‐3′) according to Hauswaldt et al. (2007). Then, genome walking strategy was employed to identify unknown sequences completely covering exon 2 and partially covering introns 1 and 2. Two sets of target‐specific primers (TSP1‐3/TSP1‐3′) were designed and used with primers DW‐ACP1‐4/DW‐ACPN/Uni‐primer from the DNA walking SpeedUp Premix Kit (Seegene) to amplify regions located at both sides of the partial exon 2 sequence in the gDNA obtained from the same frog. To get more sequence information, another two sets of target‐specific primers (2TSP1‐3/2TSP1‐3′) were used. Sequences of primers employed in genome walking are shown in Table 1.

**Table 1:** Primers used in genome walking strategy to amplify exon 2 and intron 1 and 2 regions of the MHCIIB gene from the black‐spotted frog

Sequences	Specific primers (5′→3′)	Primers from the kit (5′→3′)
Exon 2	TSP1: GACGGAGGATATCAGATATATG	DW‐ACP1: ACP1‐AGGTC
		DW‐ACP2: ACP1‐TGGTC
		DW‐ACP3: ACP3‐GGGTC
		DW‐ACP4: ACP4‐CGGTC
	TSP2: TGGAGCATCACGTTTACAATCA	DW‐ACPN: ACPN‐GGTC
	TSP3: ACAATCAGGAGGAGTTCATGT	Uni‐primer: TCACAGAAGTATGCCAAGCGA
Intron 1	TSP1′: ACATGAACTCCTCCTGATTGTA	DW‐ACP1: ACP1‐AGGTC
		DW‐ACP2: ACP1‐TGGTC
		DW‐ACP3: ACP3‐GGGTC
		DW‐ACP4: ACP4‐CGGTC
	TSP2′: TAAACGTGATGCTCCATATATC	DW‐ACPN: ACPN‐GGTC
	TSP3′: ATATATCTGATATCCTCCGTC	Uni‐primer: TCACAGAAGTATGCCAAGCGA
Intron 2	2TSP1: ATATCAGATATATGGAGCATCAC	DW‐ACP1: ACP1‐AGGTC
		DW‐ACP2: ACP1‐TGGTC
		DW‐ACP3: ACP3‐GGGTC
		DW‐ACP4: ACP4‐CGGTC
	2TSP2: GTTTACAATCAGGAGGAGTTC	DW‐ACPN: ACPN‐GGTC
	2TSP3: GGATTCTTCATTGGCATAACC	Uni‐primer: TCACAGAAGTATGCCAAGCGA
Intron 1	2TSP1′: CGTGATGCTCCATATATCTGAT	DW‐ACP1: ACP1‐AGGTC
		DW‐ACP2: ACP1‐TGGTC
		DW‐ACP3: ACP3‐GGGTC
		DW‐ACP4: ACP4‐CGGTC
	2TSP2′: TCATCATCACACTAGGGTCAC	DW‐ACPN: ACPN‐GGTC
	2TSP3′: ACATTGGCGGTATTACCTCATC	Uni‐primer: TCACAGAAGTATGCCAAGCGA

### 2.3 Screening of MHCIIB gene variations

Primers Peni‐IIB‐F (5′‐ACTCTGTATATAAGGCTGTGC‐3′) and Peni‐IIB‐R (5′‐TCTCCCTGCAGAAGATTTCA‐3′) located in exon 2 and intron 2, respectively, were used to screen for MHCIIB genetic variations in 67 black‐spotted frogs. The length of the target fragment was predicted to be 314 bp, of which 262 bp belonged to exon 2. To protect against making and propagating mistakes while copying DNA, a high‐fidelity polymerase (Ex‐Taq DNA polymerase; TaKaRa) was used to amplify the target fragment. Genotypes were identified according to the results of SSCP, heteroduplex (HD), and bidirectional Sanger sequencing analyses. Homozygous and heterozygous genotypes were distinguished using SSCP‐HD analysis. Products from homozygous samples were purified and sequenced directly. Products from heterozygous samples were purified, sequenced, and cloned into the pMD18‐T vector (TaKaRa); the resulting clones were sequenced. Bidirectional Sanger sequencing was performed by Invitrogen. The results were verified by comparing band patterns of the amplified fragments with those of mixed clones. To confirm the genotype of each sample, the procedures described above were repeated three times. New alleles were identified based on the location of the variation. Sequences were regarded as new alleles when the variation was present in exon 2.

### 2.4 Data analysis

Nucleotide and amino acid sequences were aligned in Clustal X (Larkin et al., 2007). The rates of synonymous (*d*_S_) and nonsynonymous (*d*_N_) nucleotide substitutions were calculated by the Nei and Gojobori's method with the Jukes–Cantor correction using MEGA7.0 (Kumar, Stecher, & Tamura, 2016). Antigen‐binding sites (ABSs) were inferred based on the codons involved in pathogen binding in humans (Brown et al., 1993). Recombination events were identified using RDP 4.71 supported by a series of nonparametric recombination detection methods including RDP, GENECONV, BootScan, MaxChi, Chimaera, SiScan, PhylPro, LARD, and 3Seq (Martin, Murrell, Golden, Khoosal, & Muhire, 2015). Two approaches were adopted to assess natural selection at the sequence level. First, the selection for all codons, ABS, and non‐ABS regions was assessed by calculating the *d*_N_ */d*_S_ ratio with a *Z*‐test in MEGA7.0 (Kumar et al., 2016). Second, the selection for each codon was estimated using three methods (SLAC, FEL, and IFEL) available in Data Monkey online (Delport, Poon, Frost, & Pond, 2010). Allele frequency, expected heterozygosity (*H*e), observed heterozygosity (*H*o), and Hardy–Weinberg (HW) equilibrium of the MHCIIB locus in the three geographic populations were calculated using Arlequin ver 3.5.2.2 (Excoffier & Lischer, 2010), which was also employed to estimate genetic differentiation among the populations based on FST values (Excoffier & Lischer, 2010). Phylogeny of MHCIIB alleles from *P*. *nigromaculatus*,* P*. *lessonae* (JN412618.1, JN412619.1, JN412620.1, JN412621.1), *P*. *kurtmuelleri* (JN412617.1), *Rana yavapaiensis* (JN638858.1, HQ025942.1, KU877086.1, KU877060.1, KU877052.1, JN638873.1, JN638861.1), *Rhacophorus omeimontis* (KT276475.1, KR232072.1, KT276494.1, KT276450.1, KT276472.1), *Leiopelma hochstetteri* (KP893052.1, KP893020.1, KP893031.1, KP893006.1, KP893036.1), *Alligator sinensis* (AY491429.1), and *Nipponia nippon* (AY572969.1) was estimated based on the Bayesian inference (Ronquist et al., 2012). The most likely substitution model was first created using a likelihood framework implemented in jModelTest 2.1.3 (Darriba, et al., 2012) which tested 88 different substitution models. As a result, TPM3 + G was estimated as the most likely model based on the Akaike information criterion (AIC). Bayesian analysis was performed using Mrbayes 3.2.6; four heated and one cold Markov chains were run for 1,000,000 generations, sampling each 100 generations, until stationary distribution indicated by an average standard deviation <0.01 was reached (Ronquist et al., 2012). The first 2,500 trees were discarded as “burn‐in”. FigTree v1.4.3 was used to display and edit the phylogenetic tree (http://tree.bio.ed.ac.uk/software/figtree/).

## 3 RESULTS

### 3.1 Identification of exon 2 and introns 1 and 2 of the MHCIIB gene

A 110‐bp exon‐2 fragment was amplified from the cDNA of the black‐spotted frog liver using primers MHC‐AN‐3F and MHC‐XEN‐M1‐R and then used to obtain a 445‐bp fragment covering 169 bp of exon 2 and 276 bp of intron 1 from the gDNA of the same frog by the first genome walking. As the fragment was too short to design primers for examining genetic variations, the second genome walking was conducted. Finally, a 1334‐bp MHCIIB fragment was obtained, which included 802 bp of intron 1, 270 bp of exon 2, and 262 bp of intron 2 (GenBank accession number: KY363525).

### 3.2 Polymorphism of the MHCIIB gene

**Figure 2:**
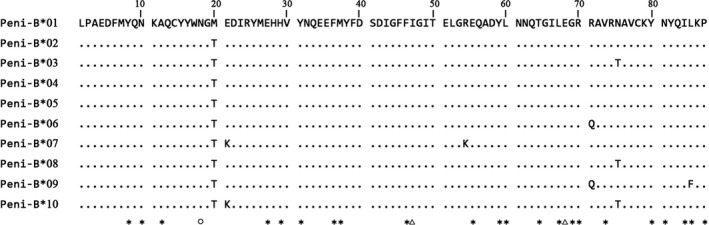
Amino acid sequence alignment of MHCIIB alleles from the black‐spotted frog. Purifying selection‐affected sites identified by all three methods (SLAC, FEL, and IFEL) or only one method (FEL) are marked with circle and triangles, respectively. Putative ABSs were defined according to the human sequence and are marked with asterisks

Thirteen unique sequences comprising 262 bp of exon 2 and 52 bp of intron 2 were identified in 67 samples from the three frog populations. The number of sequences isolated from each sample was not more than two, indicating that the primers were locus‐specific. Because intron 2 presented a noncoding region, its variations were disregarded. Finally, the 13 sequences were confirmed as 10 alleles based on the variation in exon 2 and designated as Peni‐B*01 to Peni‐B*10, respectively (GenBank accession number: KY363525‐KY363534). By comparing the 10 alleles, it could be seen that genetic variation was very limited and mainly existed in the form of single nucleotide polymorphism. There were only nine variable sites among these alleles, accounting for 3.44% of nucleotide sequence (Table 2). After deleting one nucleic base, all the alleles could be translated into normal amino acid sequences composed of 87 residues without early termination or deletion. Identical amino acid sequences were encoded by Peni‐B*02, Peni‐B*04, and Peni‐B*05 as well as by Peni‐B*03 and Peni‐B*08 (Figure 2). Thus, the 10 alleles could be translated into a total of seven different amino acid sequences which also showed limited polymorphism, as evidenced by only six identified variable sites, accounting for 6.90% of the amino acid sequence (Figure 2, Table 2). These results are consistent with nucleotide polymorphism.

**Table 2:** Parameters of genetic diversity for the black‐spotted frog based on different markers

	Nucleotide level				Amino acid level			
	All		ABS		All		ABS	
	Number of alleles	Number of variable sites	Number of alleles	Number of variable sites	Number of alleles	Number of variable sites	Number of alleles	Number of variable sites
MHCIIB	10	9	2	1	7	6	2	1
MHCIA^a^	25	85	24	33	25	40	24	13
SSR^b^	6.33	/	/	/	/	/	/	/

a Forty samples from the Jinhua population were used. Genetic diversity was investigated using a 279‐bp MHCIA fragment comprising 15 codons coding antigen‐binding sites, which is much less than in the MHCIIB gene. (Gong et al., 2013; Zhao et al., 2013).

b Thirty‐five samples from the Jinhua population were used. Thirteen polymorphic microsatellite DNA markers were developed and characterized. The number of alleles per locus ranged from 3 to 12 with an average of 6.33. (Gong, 2012; Gong, et al., 2010).

### 3.3 Selection and recombination

The putative ABS was identified, and 22 of the 87 amino acids were assigned to the ABS according to the model proposed for DRB protein structure in humans (Figure 2). Among the six variable residues, one was found in the ABS, while the other five were located in the non‐ABS region (Figure 2, Table 2). The proportions of variable nucleotide and amino acid sites in the ABS were 1.52% and 4.55%, respectively. Variable sites were found in Peni‐B*09 which had nucleotide and amino acid compositions different from those of the other alleles in the ABS (Figure 2). For the non‐ABS region, the proportions of variable nucleotide and amino acid sites were 4.10% and 7.69%, respectively. Variable sites resulted from the differences in multiple alleles (Figure 2). Nucleotide change in the ABS presented nonsynonymous substitution (*d*_N_ = 0.004 ± 0.004, *d*_S_ = 0.000 ± 0.000), while changes in the non‐ABS region were mostly synonymous substitutions (*d*_N_ = 0.010 ± 0.005, *d*_S_ = 0.028 ± 0.018) (Table 3). For the entire fragment, the ratio of nonsynonymous to synonymous substitutions (*d*_N_/*d*_S_) across the 10 alleles was below 1 (Table 3). Analysis of selective pressure at each codon showed the existence of negatively selected amino acids; among them, position 18 was confirmed by all methods, while positions 47 and 68 were only confirmed by the FEL (Figure 2). Cumulatively, these findings suggest that in the black‐spotted frog, the MHCIIB locus may be subject to purifying selection. In addition, no recombination events were detected by four different methods, indicating that genetic recombination may not be involved in the polymorphism of this locus in the black‐spotted frog.

**Table 3:** Average rates of nonsynonymous and synonymous substitutions in the ABS and non‐ABS of the MHCIIB gene from the black‐spotted frog

Region	*N*	*d*_N_	*d*_S_	*d*_N_ */d*_S_	*p*
ABS	22	0.004 ± 0.004	0.000 ± 0.000	NC	.154
Non‐ABS	65	0.010 ± 0.005	0.028 ± 0.018	0.357	.179
All	87	0.009 ± 0.004	0.020 ± 0.013	0.450	.197

*N*, number of codons in each region; *d*, average rate of nonsynonymous (*d*_N_) or synonymous (*d*_S_) substitutions per codon ± SE; and *p*, significance of assuming positive selection (*d*_N_ > *d*_S_) or purifying selection (*d*_N_ < *d*_S_) analyzed by the *Z*‐test; NC, no calculation.

### 3.4 Divergence among different geographic populations

**Figure 3:**
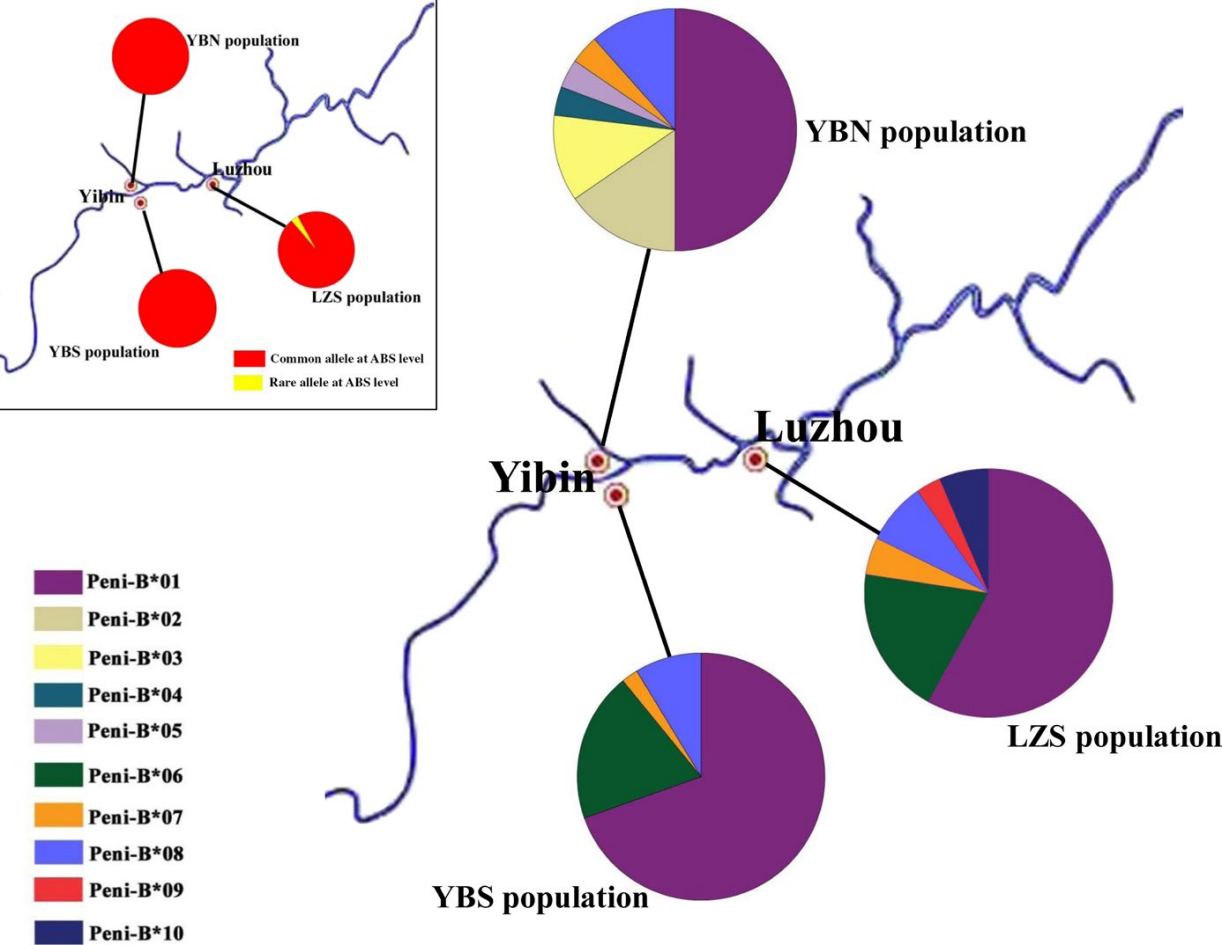
Allele distributions and relative frequencies of the MHCIIB gene in three geographic populations of the black‐spotted frog. Each allele is represented by a different color

**Figure 4:**
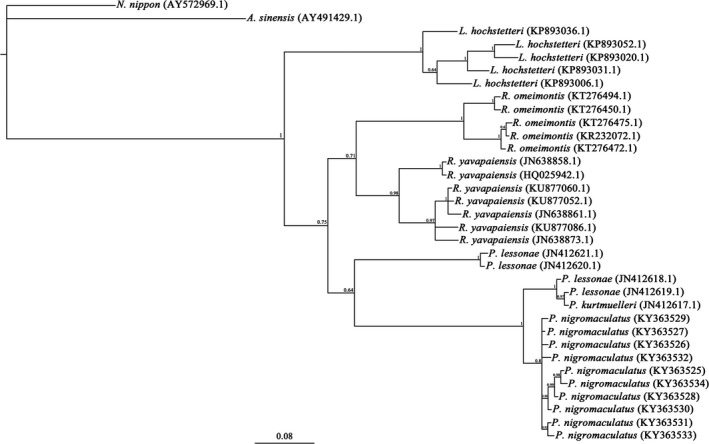
Phylogenetic tree of MHCIIB partial exon 2 from the black‐spotted frog and other species (*P. lessonae, P. kurtmuelleri, R. yavapaiensis, R. omeimontis, L. hochstetteri, A. sinensis, and N. nippon*). The credibility value for each node is shown above the branches. Each branch length is proportional to estimated DNA sequence changes (0.08 substitutions per site)

Four, seven, and six alleles were found in the YBS, YBN, and LZS populations, respectively (Figure 3, Table 4). Four specific alleles (Peni‐B*02, Peni‐B*03, Peni‐B*04, and Peni‐B*05) existed in the YBN population, which was separated from the other two populations by the Yangtze River (Figure 3, Table 4). Alleles Peni‐B*01, Peni‐B*06, Peni‐B*07, and Peni‐B*08 found in the YBS population were common and could be observed in LZS population entirely and in YBN population partly (Figure 3, Table 4). Two specific alleles, Peni‐B*09 and Peni‐B*10, were found in the LZS population (Figure 3, Table 4). The FST values among the three populations ranged from −0.00185 to 0.06856, and the difference between the YBS and YBN populations was significant (0.06856, *p *<* *.05) (Table 5), indicating that the genetic structure of the YBS population differed from that of the YBN population. However, the difference between the YBS and LZS populations was not statistically significant (−0.00185, *p *>* *.05; Table 5). To further explore the relationships among the alleles, a phylogenetic tree was constructed using the Bayes method (Figure 4). The results indicated that the alleles did not group according to geographical location. The common alleles did not form a cluster, but intertwined with some specific alleles. Furthermore, all black‐spotted frog alleles were separated from MHCIIB alleles of other amphibians (Figure 4).

**Table 4:** Allele frequencies and heterozygosity of the MHCIIB gene in three geographic populations of the black‐spotted frog

Allele	Population		
	YBS	YBN	LZS
Peni‐B*01	0.6957	0.5000	0.5806
Peni‐B*02	/	0.1538	/
Peni‐B*03	/	0.1154	/
Peni‐B*04	/	0.0385	/
Peni‐B*05	/	0.0385	/
Peni‐B*06	0.1957	/	0.1935
Peni‐B*07	0.0217	0.0385	0.0484
Peni‐B*08	0.0870	0.1154	0.0806
Peni‐B*09	/	/	0.0323
Peni‐B*10	/	/	0.0645
*H*_O_	0.39130	0.53846	0.58065
*H*_E_	0.48019	0.72308	0.62136
*p*	.12865	.03176	.03905

**Table 5:** Pairwise FST values (below the diagonal) and the significance of their differences (above the diagonal) determined for three geographic populations of the black‐spotted frog

	YBS	YBN	LZS
YBS	0.00000	**+**	−
YBN	0.06856	0.00000	−
LZS	−0.00185	0.04045	0.00000

Significance of FST values is indicated as “+” (*p *<* *.05) and “−” (*p *>* *.05).

## 4 DISCUSSION

### 4.1 Characteristics of the MHCIIB gene in the black‐spotted frog

In this study, we successfully identified a MHCIIB gene in the black‐spotted frog. The MHC fragment was amplified by step‐wise genome walking strategy; then, locus‐specific primers were designed to screen frog populations based on the acquired sequence. As a result, no more than two alleles were amplified in each sample, indicating that only one locus was isolated from the black‐spotted frog. Thus, in this study, we developed a locus‐specific genotyping technique, providing guidance for characterization of MHC‐encoding genes in other amphibians. The identified locus is evidently active in the frog immune system as it could be isolated from the cDNA sequences and translated into amino acid sequence without early termination or deletion. Compared to the MHCIA gene and SSR, this locus presented very limited polymorphism both in the nucleotide and amino acid composition (Figure 2, Table 2). Limited polymorphism might be region‐specific, as populations from three geographical locations were used. In addition, other MHCIIB loci might exist in the genome of the black‐spotted frog, because at least two MHCIIB loci have been found in *P. lessonae*, the other member of the *Pelophylax* genus (Marosi, et al., 2014) (Figure 4). The specificity of the MHCIIB gene to the *Pelophylax* genus was proven by allele clustering (Figure 4). Trans‐species polymorphism among closely related species is typical for MHCIIB alleles, but none of Peni‐B alleles mixed with the alleles from other *Pelophylax* species (Figure 4), probably because very few alleles in the genus could be used for phylogenetic analysis. Therefore, the identification of the MHCIIB locus and alleles in this study has laid a foundation for future studies on adaptive evolution of the *Pelophylax* species.

### 4.2 Purifying selection

Positive, diversifying, and purifying selection are three basic types of natural selection acting at the gene level (Harris & Meyer, 2006). It is established that MHC‐encoding genes are highly polymorphic because of diversifying selection driven by pathogens (Consuegra et al., 2013; Hauswaldt et al., 2007; Shu et al., 2013; Yu et al., 2014). However, in the present study, we found that the identified locus was under purifying selection. First, the number of variable nucleotide and variable amino acid sites was quite limited. Even in the ABS, only one variable nucleotide site out of 66 (1.52%) and only one variable amino acid site out of 22 (4.55%) were found (Figure 2, Tables 2 and 3). Second, the number of nonsynonymous substitutions was less than that of synonymous substitutions for all the codons. Although it was the opposite situation for the ABS, the difference was caused by one base variation in Peni‐B*09. Accordingly, the result of natural selection acting at the ABS was uncertain (Figure 2, Tables 2 and 3). Third, the existence of negatively selected sites was revealed by analyzing selection pressure at each codon (Figure 2). It is a rare case that a MHC gene is under purifying selection, and functional activity of the encoded proteins in the immune system of the black‐spotted frog should be analyzed to explain this phenomenon. It is possible that these proteins are responsible for the recognition and presentation of a class of special finite antigens or act as molecular chaperones (Adams & Luoma, 2013; Jensen, 2007).

### 4.3 Divergence caused by physiographic factors

Physiographic factors such as big rivers and mountains can influence the genetic structure of different species, especially those lacking dispersal ability (Bushar et al., 2014; Lau, et al., 2014). As expected, substantial differences were found in genetic structure among the three geographic populations (Figure 3, Tables 4 and 5). There was a significant difference between geographically separated YBS and YBN populations (*p *<* *.05; Figure 3, Tables 4 and 5), while no difference was detected between YBS and LZS populations (*p *>* *.05), indicating slight genetic difference between the latter (Figure 3, Table 5). Evidently, the geographical barrier presented by the Yangtze River which separates the YBN population from the other two plays a crucial role in shaping the genetic structure of the black‐spotted frog. However, the phylogenetic tree revealed that the alleles did not cluster by geographical location and that common and special alleles were mixed together (Figure 4), which may be related to the repeated spreading and contraction of black‐spotted frog populations caused by climate changes during middle‐late Pleistocene and last interglacial and glacial periods (Zhang, Yan, Zhang, & Zhou, 2008). The genetic structure of black‐spotted frog populations can be a result of the interaction of physiographic and climatic factors but also may be affected by ancestral polymorphism.

## ACKNOWLEDGMENTS

This work was supported by a grant from the National Natural Science Foundation of China (No. 31170349), a special grant from the State Forestry Administration, and the Fundamental Research Funds for the Central Universities (2016FZA6002) of the P. R. China.

## CONFLICT OF INTEREST

None declared.

## AUTHOR CONTRIBUTION

Hong‐Yi Liu, Fei Xue, and Jie Gong conducted experiments. Hong‐Yi Liu, Qiu‐Hong Wan, and Sheng‐Guo Fang analyzed the data and wrote the manuscript. Jie Gong collected the samples. Sheng‐Guo Fang conceived and designed the study. All authors read and approved the revision of the manuscript.
